# Nutritional, phytochemical, and in vitro anticancer potential of sugar apple (*Annona squamosa*) fruits

**DOI:** 10.1038/s41598-021-85772-8

**Published:** 2021-03-18

**Authors:** Mohamed Gamal Shehata, Marwa Muhammad Abu-Serie, Nourhan Mohammad Abd El-Aziz, Sobhy Ahmed El-Sohaimy

**Affiliations:** 1 Department of Food Technology, Arid Lands Cultivation Research Institute, City of Scientific Research and Technological Applications (SRTACITY), P.O. Box 21934, New Borg El-Arab City, Alexandria Egypt; 2 Department of Medical Biotechnology, Genetic Engineering and Biotechnology Research Institute, City of Scientific Research and Technological Applications (SRTACITY), P.O. Box 21934, New Borg El-Arab City, Alexandria Egypt; 3 Department of Technology and Organization of Public Catering, Institute of Sport, Tourism and Service, South Ural State University, Chelyabinsk, Russia 454080

**Keywords:** Biochemistry, Biological techniques, Cancer, Molecular biology

## Abstract

In plants, Fruits and their wastes are the main sources of bioactive compounds. Currently, Annona fruits have attracted the attention of people interested in health-promoting foods due to their phytochemical content that their activities were not studied before. This study aimed to explore the potential antioxidant, antimicrobial, and in vitro anticancer activity of two cultivars *Annona squamosa* (*Annona b.* and *Annona h.*) seed, peel, and pulp. We also meausred phenolic, flavonoid, sulfated polysaccharide, tannins, and triterpenoids. Polyphenol identification was determined using RP-HPLC. Results of the antioxidant activity revealed that the highest activity was observed for *Annona h.* seed extract using DPPH and ABTS assays with IC_50_ 6.07 ± 0.50 and 9.58 ± 0.53 µg/ml, respectively. The antimicrobial activity against various pathogenic strains revealed that the peel extracts of both *Annona b.* and *Annona h.* exhibited the best antimicrobial activity. We also assessed the IC_50_ values for anticancer activity in all six *Annona b.* and *Annona h* samples against four cancer cell lines colon (Caco-2), prostate (PC3), liver (HepG-2), and breast (MCF-7) using MTT assay. *Annona b.* and *Annona h* seed extracts had the lowest IC_50_ values for four cancer cell lines with 7.31 ± 0.03 and 15.99 ± 1.25 for PC-3 and MCF-7, respectively. Both seed extracts, *Annona b.* and *Annona h.*, showed significantly down-regulated mRNA expression of Bcl-2 and up-regulated p53 in all treated cell lines. Apoptosis was evaluated using nuclear staining, flow cytometric analysis, and immunohistochemistry of the proliferation marker (Ki-67). Additional studies are required to characterize the bioactive compounds responsible for the observed activities of Annona seed and determine its mechanism as an anticancer drug.

## Introduction

The sugar apple family (*Annonaceae*) is a pan-tropical family that contains approximately 130 genera and 2300 species of trees and shrubs^1^. In the wild, plants from this family produce a large range of exotic fruits and have distinctive varieties, such as graviola fruit (*Annona muricata*), araticum-do-cerrado or marolo (*Annona crassiflora*) and conde fruit (*Annona squamosa*)^2^. *Annona squamosa* is belong to the family Annonaceae. The fruit of this plant is called Keshta in Egypt, although it has been called a sugar apple by the English-speaking community. In America and the West Indies areas, this plant (*A. squamosa*) can be cultivated. This plant is being commercially grown worldwide because of recent exposure to its medicinal properties^3,4^. Many ethnic groups use squamosa for the treatment of many chronic illnesses^5^. Many Annonaceae members are used as anti-cancer^6^, anti-inflammatory^7^, anti-HIV^8^, and anti-bacterial and anti-fungal^9^ therapies in conventional medicine.

Cancer is the second mortality reason after cardiovascular disease^10^. It was expected that the number of dies from cancer will increase to 14.6 million by 2035^11^. Oxidative stress and reactive oxygen species (ROS) are considered as a strong risk factor that trigger inflammatory response and cancer^12^. Homeostasis of ROS in the human body kept balanced by non-enzymatic and antioxidant manipulators. Imbalance between antioxidants and oxidant modifiers causing damage of cell biomolecules such as lipids, proteins, and DNA^13^. ROS attack the nucleotide of cell DNA causing mutations and consequently cancer, tumor suppressor genes mutations such as p53 mutation is an example^14^. Apoptosis is a program cell death, furtiveness of apoptosis resulting in uncontrolled cell division and cancer^15^. Decision of cell division and apoptosis determined by the balance of proto-oncogene Bcl-2 and tumor suppressor p53 proteins which are involved in many biochemical pathways related to cancer progression^16^. Nowadays, intensive research efforts are presented to find a new cancer drug can target save apoptosis in cancer cells^17^. The diversity of cancer types and leading causes increase the demands for new treatment. In United States 1,806,590 new cancer cases and 606,520 cancer deaths only in 2020^18^. At the same time, a new type of natural compound is being discovered every day. In 2006, approximately 50,000 of the newly natural molecules were discovered, this number were grow up to around 326,000 in 2014. Among them, a round 195,000 were biologically active^19^. However, about 170,000 compounds were toxic. Conventional medicinal herbs and other plants regarded as a huge source of pharmacologically active compounds^20^. Due to their alkaloid content, Annona plants have been used previously as antibacterial treatment for many related diseases like bacterial caused ulcers, dysentery, and boils^21^. There have been no previous studies on the biological activities of the different parts (peel, seeds, and pulp) of Egyptian Annona spp. Therefore, the current study explores the phytochemical analysis of Annona cultivated under Egyptian climate conditions as well as elucidation of the antioxidant, antimicrobial, and anticancer activities of its different parts (peel, seed, and pulp) in vitro.

## Results and discussion

### Proximate composition analysis

The proximate compositions of different parts of *Annona* spp. were presented in Table 1. The obtained results showed that the moisture content of the pulp of the two species (*Annona b.* and *Annona h*.) varied from 81.66 ± 1.35 to 83.40 ± 0.61%, respectively. The highest protein content was recorded in *Annona b.* peels with a concentration of 3.30 ± 0.16%. The fat content ranged from 0.96 ± 0.23 to 29.21 ± 1.12% for *Annona b. pulp* and *Annona b.* seeds, respectively*.* The carbohydrate content ranged from 93.77 ± 1.78 to 94.36 ± 1.26%; the crude fiber content ranged from 66.64 ± 3.92 to 94.36 ± 1.26% for *Annona b. pulp* and *Annona b.* pulp, respectively. Finally, the ash content varied from 2.12 ± 0.19 to 3.14 ± 0.28%. Diversity in the gross chemical composition could be attributed to several factors, such as cultivar, ripening stage of fruit, climatic conditions, fertilization and irrigation system, and harvesting time^22^. It has been reported that the protein content of *Annona muricata* (L.) peels and seeds were 1.56 ± 0.01 and 2.73 ± 0.10%, respectively^23,24^, which is less than current findings in peels and much more in seeds in both species. At the same time, other studies showed that the protein content of *Annona crassiflora* pulp was 1.38 ± 0.01%^25^, which was less than the results of the present investigation (2.133 ± 0.33%). *Annona b.* and *Annona h.* contained all essential minerals and, *Annona b.* samples had the highest mineral concentrations, as shown in Table 1*.* On the other hand, *Annona b.* pulp showed the highest concentration of all measured minerals except potassium, which found to be high in *Annona b.* peel (115.78 mg/kg). *Annona b.* pulp showed high concentration of calcium, copper, iron, magnesium, manganese, sodium, phosphorus, and zinc with values of 84.33, 0.38, 20.73, 56.78, 0.33, 45.24, 55.20, and 0.31%, respectively (Table 1). An adequate concentration of calcium was observed in the pulp of *Annona b.* (84.33 mg/kg) and the seed of *Annona h.* (75.11 mg/kg), which represents approximately 8.4% and 7.5% of the recommended daily intake (1000 mg)^26^. The pulp of *Annona b.* showed a considerable concentration of iron (20.72 mg/kg) that represents approximately 133% of the daily requirement (15 mg). Similarly, both *Annona* species exhibited high concentrations of potassium. The findings of the current investigation emphasized the adequate concentrations of essential minerals in both Annona species such as calcium that is important for healthy bone and teeth. Iron plays a crucial role by preventing anemia and potassium deficiency. Essential minerals are also responsible for the maintenance of an optimal pH balance and blood pressure regulation in the human body.

**Table 1:** Proximate chemical composition of different parts for *Annona b.* and *Annona h.*

g/100 g dry weight (%)	*Annona b.*			*Annona h.*		
	Peel	Pulp	Seed	Peel	Pulp	Seed
Moisture	3.50 ± 0.31^d^	81.66 ± 1.35^b^	3.92 ± 0.19^ cd^	5.36 ± 0.23^c^	83.40 ± 0.61^a^	4.39 ± 0.14^ cd^
Protein	3.30 ± 0.16^a^	2.13 ± 0.33^bc^	2.25 ± 0.28^bc^	2.99 ± 0.47^ab^	1.97 ± 0.34^c^	1.90 ± 0.56^c^
Fat	7.78 ± 0.46^c^	0.96 ± 0.23^d^	29.21 ± 1.12^a^	6.83 ± 0.20^c^	1.55 ± 0.32^d^	24.83 ± 1.21^b^
Carbohydrate	86.75 ± 1.94^a^	93.77 ± 1.78^c^	66.64 ± 3.92^b^	87.33 ± 1.91^a^	94.36 ± 1.26^c^	70.91 ± 3.82^b^
Fiber	9.17 ± 0.61^b^	2.66 ± 0.19^c^	32.64 ± 1.87^a^	8.50 ± 0.45^b^	1.90 ± 0.24^c^	34.10 ± 1.32^a^
Ash	2.17 ± 0.15^bc^	3.14 ± 0.28^a^	1.90 ± 0.50^c^	2.85 ± 0.24^ab^	2.12 ± 0.19^bc^	2.36 ± 0.44^bc^
**Minerals (mg/kg)**						
Ca	51.22	84.33	46.90	29.31	14.41	75.11
Cu	0.20	0.38	0.30	0.12	0.07	0.28
Fe	1.05	20.73	6.74	1.41	0.85	1.21
K	115.78	112.54	56.47	108.30	106.86	56.21
Mg	38.60	56.78	20.36	27.92	15.18	16.78
Mn	0.19	0.33	0.25	0.18	0.05	0.28
Na	32.18	45.24	9.29	29.54	13.88	7.85
P	47.67	55.20	33.30	33.32	17.10	33.49
Zn	0.22	0.31	0.43	0.27	0.10	0.64

Means ± SD (standard deviations) in the same row followed by the same letters are not significantly different (*P* < 0.05). Each data point is the average of three replications.

### Phytochemical content

**Figure 1:**
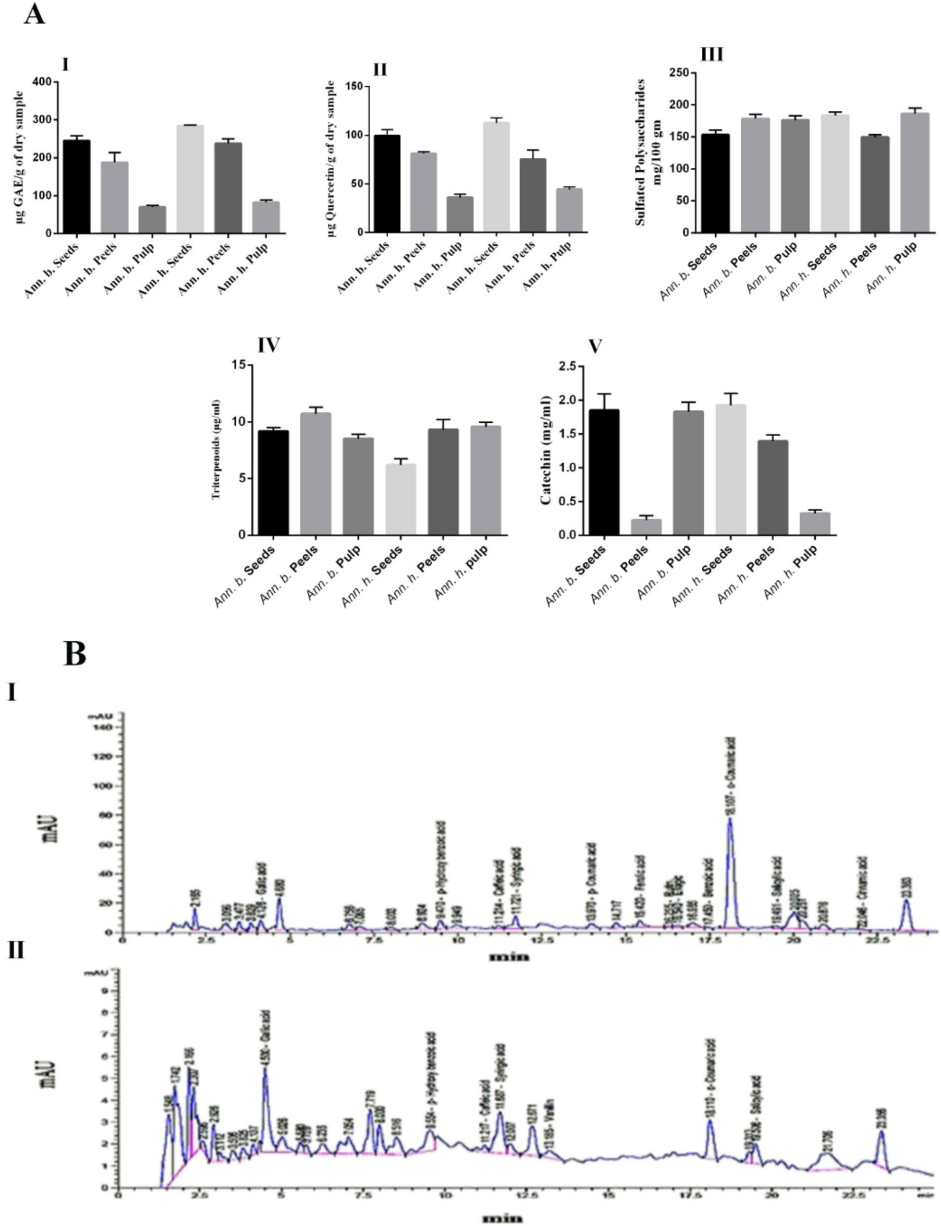
(**A**) Phytochemical content in pulp, seed, and peel of *Annona* (µg/g) (I) Total Phenolic, (II) Total Flavonoids, (III) Sulfated Polysaccharides, (IV) Triterpenoid, (V) Tannin. (**B**) Representative RP-HPLC chromatograms of phenolic compounds of seed extract of Annona sp.: (I) *Annona b.* (II) *Annona h.* Peak identifications were performed by matching retention time and UV spectra against standard compounds.

Phytochemical content in pulps, seeds, and peels of Annona are showed in Fig. 1A. The total phenolic content of the different parts ranged from 70.14 ± 3.89 to 284 ± 2.12 µg GAE/g of dry sample. The highest total phenolic content was observed in seed extracts of *Annona h*. (284 ± 2.12 µg GAE equivalent/g). The flavonoid content ranged from 36.05 ± 3.29 to 112.71 ± 4.93 µg QE equivalent/g of dry sample. The seed extract of *Annona h*. exhibited the highest flavonoid content (122.71 ± 4.93 µg QE equivalent/g), followed by the peel extract of *Annona b.* (81.27 ± 1.74 µg QE equivalent/g). The sulfated polysaccharide content ranged from 149.50 ± 3.76 to 186.37 ± 9.02 µg/g, triterpenoid content ranged from 6.20 ± 0.56 to 10.73 ± 0.57 µg/g, and tannin content of different parts of *Annona* sp*.* ranged from 0.22 ± 0.07 to 1.92 ± 0.17 µg/g of dry extract. Currently, Annona fruits have attracted the attention of people interested in health-promoting foods due to their content of bioactive compounds. Such bioactive compounds have been reported to have health benefits due to their ability to scavenge harmful free radicals^27,28^. Utilization of bioactive compounds such as natural phenolics with antioxidant activity plays a crucial role in the food industry for the formulation of health-promoting foods. The findings of the present work are comparable with those described in the literature^8,28^. It is worth mentioning that the matrices studied in the current work were approximately 1.5-fold richer than those described in the previous studies in terms of total phenolic content. Notably, this is the first study exploring the concentrations of tannins, triterpenoids, and sulfated polysaccharides in *Annona h.* and *Annona b.*, as these aspects have never been reported previously.

### Phenolic compound profile (RP-HPLC)

Phenolic compounds of the seed extracts of both *Annona b.* and *Annona h.* are shown in Fig. 1B. Results revealed that the main differences between the two extracts are based on the presence or absence of vanillin, p-coumaric acid, ferulic acid, rutin, ellagic acid, and benzoic acid and that the relative amounts of all the compounds are present. The most abundant compounds in the *Annona b.* seed extract were gallic acid, p-hydroxybenzoic acid, syringic acid, ferulic acid, ellagic acid, benzoic acid, o-coumaric acid, and salicylic acid. These compounds are by far the most abundant compounds, not only in the seed of *Annona b.* but also in the seed of *Annona h.* except for ferulic acid, ellagic acid, and benzoic acid. Cinnamic acid was detected in low quantity in *Annona b.* seed extract and was not found in *Annona b.* seed extract. Moreover, cinnamic acid derivatives (p- Coumaric acid, ferulic acid, and o- coumaric acid) were detected only in *Annona b.* seed extract at concentrations of 1.96, 5.08, and 49.02 mg/100 g, respectively. Janicke et al.^29^ reported that dietary fiber is a rich source of hydroxycinnamic acids, ferulic acid, and p-coumaric acid, all of which may contribute to its protective effect against colon cancer. Besides Pei et al.^30^ confirmed the antioxidant, anti-inflammatory, anti-mutagenic, anti-ulcer, and anticancer activities of p-coumaric acid. Also, many studies have shown that o-coumaric acid has antioxidant, and antitumor biological activity^31,32^.

### Antioxidant activity

**Figure 2:**
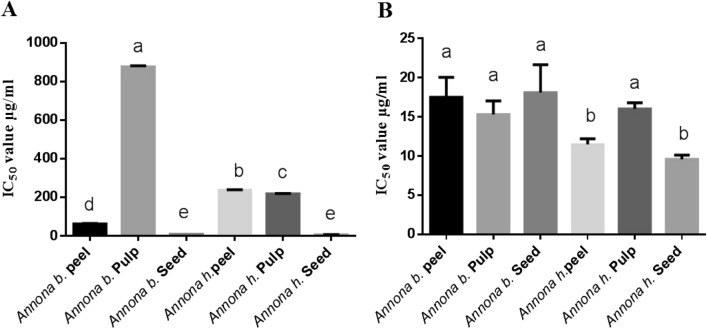
The antioxidant IC_50_ values (µg/ml) of *Annona b.* and *Annona h.* Peel, pulp, and seed. DPPH (**A**) and ABTS (**B**). Values represent means ± SD (standard deviations) for triplicate experiments. IC_50_ values (µg/ml) of ascorbic acid of DPPH and ABTS were 6.39 ± 0.04 and 8.75 ± 0.25, respectively. Values bearing the same letters showed no significant difference (*P < 0.05). The results are sorted in descending order: a < b < c < d < e.

Figure 2 shows the IC_50_ values of free radical scavenging activity of the extracts toward DPPH and ABTS. Concerning DPPH, the IC_50_ values of ascorbic acid and the seed extracts of *Annona b.* and *Annona h.* were 6.39 ± 0.04, 7.88 ± 0.28, and 6.07 ± 0.50 µg/ml, respectively, and the peel extract of *Annona b.* was 61.78 ± 3.16 µg/ml. This finding indicates that the seed extracts exhibited the highest antioxidant capacity; this may be attributed to the greater accumulation of polyphenols and flavonoids in the seed than in other parts of the plant. Regarding ABTS, IC_50_ values were arranged in ascending order as follows; *Annona b.* seed, *Annona h.* peel, and *Annona b.* pulp, followed by *Annona h.* pulp, then *Annona b.* peel. The seed parts of both species exhibited the highest scavenging activity against both DPPH and ABTS free radicals (Fig. 2).

The results revealed the potency of the *Annona* extracts as an excellent antioxidant. It is well known that antioxidants play a crucial role in the prevention of cancer and other oxidative stress related diseases^33^. Albuquerque et al.^34^ reported the antioxidant activity of *Annona cherimola* Mill. pulp, peel, and seed, had IC_50_ values of 452 ± 0.01, 330 ± 0.00, and 319 ± 0.08 µg/ml, respectively. These results were relatively high compared to our results for *Annona squamosal*, indicating that *Annona h.* and *Annona b* have high antioxidant activity compared to other reported Annona species. Numerous studies revealed the antioxidant activity for phenolic compounds and their ability to scavenge free radicals that disrupt biological molecules such as DNA, proteins, and lipids^35^.

### Antimicrobial activity

The results of the antimicrobial assay of the peel, seed, and pulp extracts of the two Annona spp. against six pathogenic microorganisms are shown in Table 2. *E. coli, B. subtilis, C. albicans, K. pneumoniae, S. senftenberg,* and *S. aureus* were used to test pathogenic microorganisms due to the prevalence of these microbes as foodborne pathogens. The peel extract of *Annona h.* exhibited excellent activities against *K. pneumoniae* ATCC 12296 and *S. senftenberg* ATCC 8400, with inhibition zones of 14.70 ± 1.07 mm and 16.10 ± 1.25 mm, respectively. This study is the first investigation to survey the antimicrobial activity of the peel, seed, and pulp extracts of *Annona b.* and *Annona h.,* while many investigations have evaluated the antimicrobial activity of *Annona Linn*. leaf extracts^36,37^. The highest antimicrobial activity of *Annona b.* seed extract was observed against *B. subtilis* DB 100 with an inhibition zone of 12.50 ± 1.35 mm. Chandrashekar and Kulkarni^38^ reported that the leaf extract of *Annona Linn*. has antimicrobial activity against Salm*onella typhi, S. aureus, Pseudomonas aeruginosa,* and *B. subtilis*. Generally, antimicrobial potential might be attributed to the polyphenol compounds present in the plant extracts^39–41^. Overall, the peel and seed extracts exhibited better antimicrobial activities than the pulp extracts, which might be due to the presence of active ingredients at higher concentrations in the seed and peel than in the pulp. Annona extracts (peel and seed) with high antimicrobial activity could be used to develop some functional food products to increase their shelf-life and boost the overall health benefits.

**Table 2:** Antimicrobial activity of peel, seed, and pulp extracts of *Annona b.* and *Annona h.*

Pathogens microorganisms	Diameter of inhibition zone (mm)					
	*Annona b.*			*Annona h.*		
	Peel	Pulp	Seed	Peel	Pulp	Seed
*Escherichia coli* BA 12296	11.53 ± 1.07^b^	5.50 ± 0.86^d^	9.50 ± 0.50^c^	13.16 ± 1.19^a^	6.36 ± 0.90^d^	11.10 ± 0.36^b^
*Bacillus subtilis* DB 100 host	13.93 ± 0.81^a^	10 ± 0.2^c^	12.50 ± 1.35^b^	12.06 ± 0.30^b^	7.40 ± 0.52^d^	9.83 ± 0.77^c^
*Candida albicans* ATCC MYA-2876	10.10 ± 0.65^ab^	7 ± 0.68^c^	9.53 ± 0.75^b^	11.10 ± 0.50^a^	5.03 ± 0.58^d^	9.06 ± 0.30^b^
*Klebsiella pneumoniae* ATCC12296	14.26 ± 1.07^a^	8.70 ± 0.81c	10.33 ± 0.41^b^	14.70 ± 1.07^a^	8 ± 0.50^c^	10.56 ± 0.60^b^
*Salmonella senftenberg* ATCC 8400	15.36 ± 0.85^a^	8.36 ± 0.32^c^	12.30 ± 1.05^b^	16.10 ± 1.25^a^	5.30 ± 1.11^d^	8 ± 0.86^c^
*Staphylococcus aureus* NCTC 10788	9.76 ± 0.92^a^	5.16 ± 1.25^bc^	6.50 ± 0.50^b^	9.33 ± 1.04^a^	4.70 ± 0.81^c^	6.40 ± 0.69^b^

### In vitro* cytotoxicity and anticancer activity of different Annona h. and Annona b. parts*

**Figure 3:**
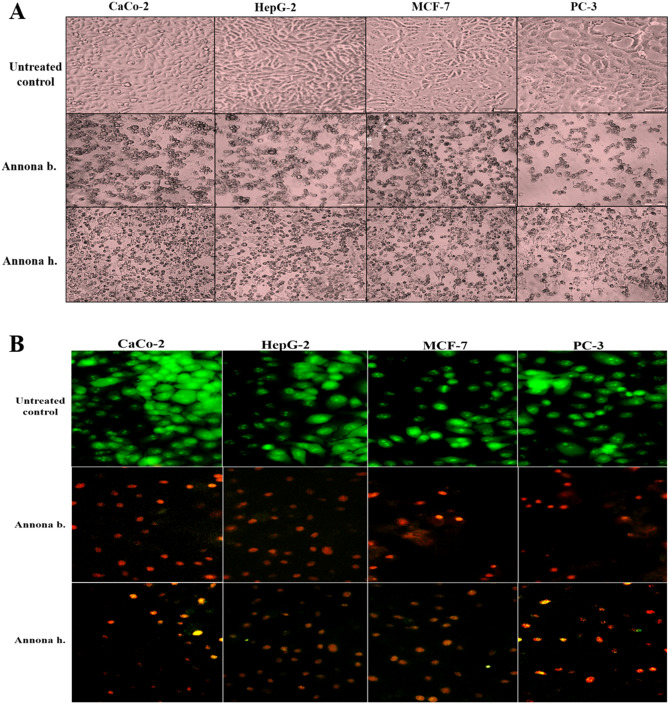
Morphological alterations of human cancer cell lines after treatment with the most active extract (seed) of both Annona species using (**A**) phase-contrast microscope and (**B**) fluorescence microscope after staining with ET/AO dye (green, yellow, and orange-red fluorescence nuclei refer to viable, early apoptotic, and late apoptotic cells, respectively). (CellSens Dimension 1.12; https://www.olympus-lifescience.com/en/software/cellsens/).

**Figure 4:**
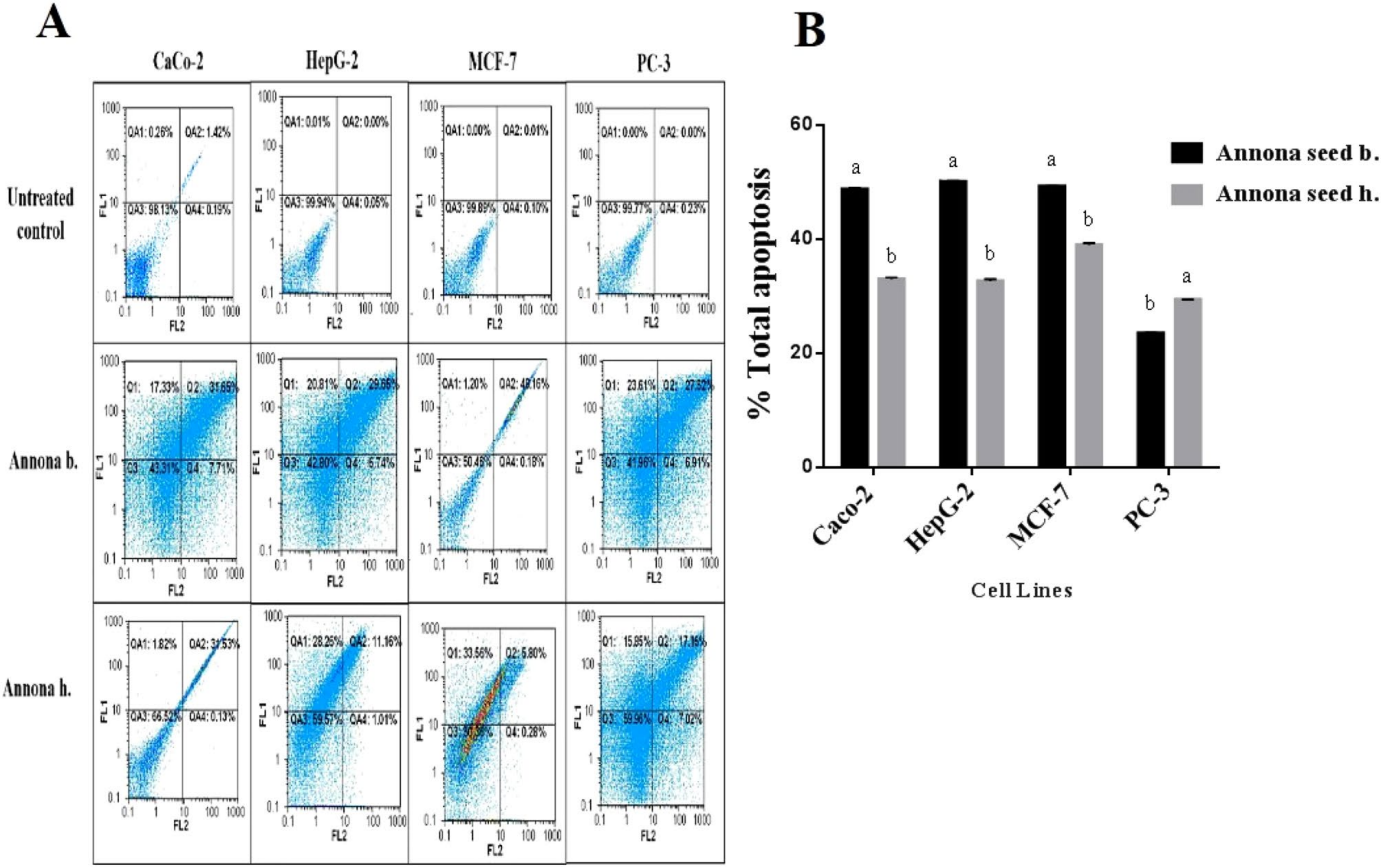
Quantitative detection of the apoptotic effect of seed extracts of Annona species in human cancer cell lines (**A**) Annexin V/Propidium Iodide (PI) flow cytometry charts with (**B**) histograms of total percentage of apoptotic cell populations, results are presented as mean ± SE (n = 3). Different letters for the same cell line are significantly different at *p* < 0.05.

The safety of different parts of Annona on normal human lung fibroblast cells (WI-38 cell line) represented by the EC_100_ values. The results recorded that the extracts of seed, peel, and pulp of Annona are safe in the range of 654.26 ± 31.50, 551.35 ± 9.70, and 552.49 ± 3.33 µg/ml, respectively for *Annona b.*, and 501.25 ± 0.43, 608.89 ± 6.36, and 493.22 ± 0.93 µg/ml, respectively for *Annona h.* The safety of an anticancer drug is one of the most important concerns of the physician in the treatment of cancer patients. The anticancer activity expressed as IC_50_ (concentration of the studied extracts that caused 50% cell death) is usually used to represent the strength and sensitivity of the drug on cancer cell lines. Table 3 demonstrates that seed extract of *Annona b.* had the highest anticancer effect against the four studied cancer cell lines (Caco-2, HepG-2, MCF-7, and PC-3). However, there was no significant difference recorded between the IC_50_ values of the seed extract from *Annona b.* and *Annona h.* for four cancer cell lines. There was no detectable anticancer efficiency of pulp and peel extracts of *Annona h.* on HepG-2 and PC-3 cells as well as peel extract of *Annona h.* against MCF-7 cells (Table 3). The most active extract (seed) of both Annona species caused a collapse in spindle shape with cellular shrinking in the treated human cancer cell lines. This obvious morphological alteration was more severe in the seed extract of *Annona b.* than that of *Annona h.* (Fig. 3A). These morphological signs of cell death were detected by fluorescence after dual staining of the treated cancer cells with ethidium bromide and acridine orange (EB/AO) as shown in Fig. 3B. Nuclei of viable, healthy cells can only be stained with AO, and they appeared with green fluorescence, but early apoptotic cells exhibit a bright green to yellow, and late apoptotic cells have bright orange to red. We found that most cell nuclei of the seed extract of *Annona b*-treated cancer cells exhibited a reddish-orange fluorescence, while *Annona h*-treated cells demonstrated a yellowish-orange fluorescence (Fig. 3B). This result was confirmed by quantitative detection of the apoptosis percentage as a response to *Annona b.* seed and *Annona h.* seed extract treatment on HepG-2, Caco-2, and MCF-7 cell lines using annexin V/propidium iodide (PI)-flow cytometric analysis (Fig. 4A,B). Results revealed a significant elevation in total apoptosis percentages (> 48%) in the *Annona b.* seed extract-treated Caco-2, HepG-2, MCF-7, and PC-3 cells in comparison with those treated by *Annona h.* seed extract (< 39%). The most affected cell lines were PC-3 (51.27 ± 0.1%) and HepG-2 (50.14 ± 0.32%), followed by MCF-7 (49.30 ± 0.07%) and Caco-2 (48.81 ± 0.16%) after *Annona b.* seed extract treatment. On the other hand, the most affected cell lines were MCF-7 (38.99 ± 0.37%) and Caco-2 (33.05 ± 0.3%) followed by HepG-2 (32.67 ± 0.33%) then PC-3 (29.43 ± 0.035%) after *Annona h.* seed extract treatment.

**Table 3:** IC_50_ (µg/ml) of different extracts of *Annona b*. and *Annona h*. against proliferation of human cancer cell lines.

Cells	*Annona b*.			*Annona h*.		
	Peel	Pulp	Seed	Peel	Pulp	Seed
Caco-2	30.69 ± 0.35^b^	45.05 ± 5.80^b^	11.55 ± 1.04 ^a^	44.68 ± 2.80^b^	34.24 ± 1.60^b^	12.81 ± 0.02^a^
HepG-2	15.28 ± 0.67^b^	27.56 ± 2.10^c^	7.99 ± 0.21^a^	> 200	> 200	10.21 ± 1.78^a^
MCF-7	15.17 ± 0.17^a^	27.82 ± 5.50^b^	14.34 ± 0.35^a^	> 200	42.39 ± 0.64^c^	15.99 ± 1.25^a^
PC-3	14.36 ± 0.35^b^	22.65 ± 1.32^c^	7.31 ± 0.03^a^	> 200	> 200	10.96 ± 0.64^a^

Results are presented as Mean ± SE (n = 3). Different letters in the same row are significantly different at p < 0.05; IC50, concentration of the studied extracts that caused 50% viability for tested cells.

Natural products are still important sources to discover new anticancer drugs^42^. The National Cancer Institute has collected about 35,000 plant samples from 20 countries and has screened around 114,000 extracts for anticancer activity^43^. Aidy et al.^44^ reported the anticancer potential activity of *Annona muricata* against breast, prostate, pancreatic, liver, lung, and colon cancers. Also, the anticancer properties of graviola (*Annona muricata*) were reported by Islam et al.^45^. Another report mentioned the anticancer potential of *Annona muricata* L. leaf extract^46^. *Annona b.* and *Annona h.* seed extracts and its polyphenols may be potentiating their anticancer activity. Polyphenols from diet have previously been documented to interfere many of biochemical pathways involved in cancer progression^47^. Furthermore, polyphenols may act as immune system booster, as well as help in living cells protection from ROS damage.

Many clinical trials of polyphenols treatments confirming the protective mechanism of them due to variations in dose, timing, and other conflicting factors^48^. Thus, this in vitro study aimed to evaluate the anticancer potential of two Egyptian Annona species fruits and their byproducts as a first step for use in medical applications.

### Relative gene expression of Bcl-2 and p53 in different cell lines treated by Annona seed

**Figure 5:**
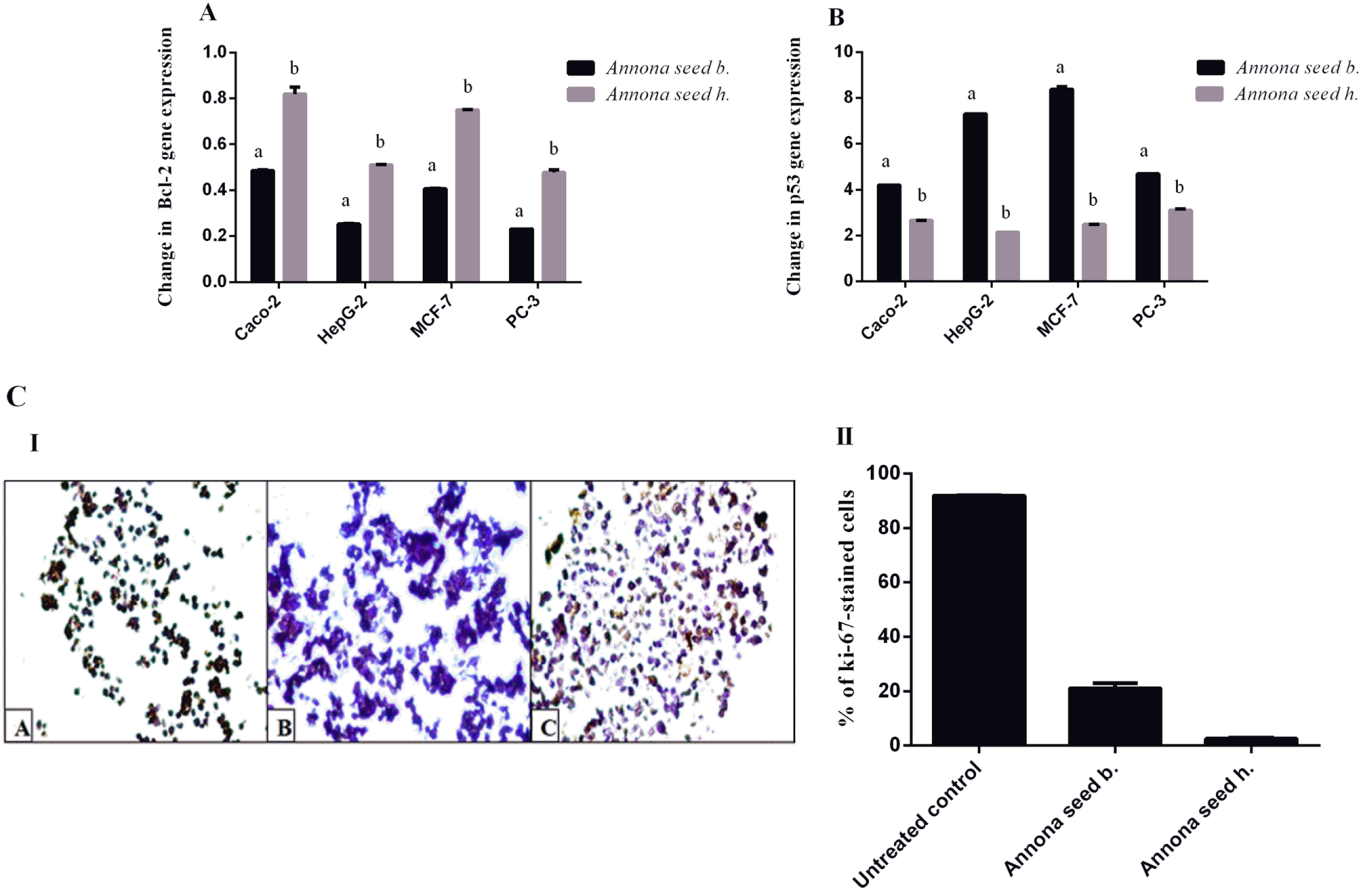
Effect of the studied *Annona b.* seed extract and *Annona h.* seed extract on relative gene expression of (**A**) Bcl-2 and (**B**) p53 in different cell lines as well as (**C**) Ki-67 immunostaining in the treated HepG-2 cells illustrated as (I) Microscopic images with (II) Percentage of Ki-67 immunostained cells. Results are presented as mean ± SE (n = 3). Different letters for the same cell line are significantly different at *p* < 0.05. (CellSens Dimension 1.12; https://www.olympus-lifescience.com/en/software/cellsens/).

The tumor suppressor p53 and proto-oncogene Bcl-2 are critical mediators of apoptosis and carcinogenesis^16^. Both studied seed extracts of *Annona b.* and *Annona h.* significantly down-regulated the expression of Bcl-2 and up-regulated p53 mRNA expression in PC-3, HepG-2, Caco-2, and MCF-7 treated cell lines. *Annona b.* seed extract suppressed Bcl-2 expression (0.23–0.48) in the treated human cancer cell lines by a higher extent than *Annona h* (0.47–0.82) as shown in Fig. 5A. Furthermore, the seed extract of *Annona b.* enhanced the expression of p53 by more than fourfold, which was higher than in *Annona h*-treated cancer cells (2.15 to 3.10-fold) (Fig. 5B). Bcl-2 itself is an anti-apoptotic gene that prevents the initiation steps of apoptosis and programmed cell death^49^. These results suggested that *Annona b.* seed extract had a higher potency against apoptosis induction than *Annona h.* seed extract in all tested cancer cell lines. Wild type p53 is an important regulatory protein in the induction of apoptosis following DNA damage induced by anticancer drugs^50^. This tumor suppressor protein leads to the arrest of growth of viable cells in the G1 phase or apoptosis^51^. Further in vitro studies are required to determine the detailed anticancer mechanism of *Annona b.* seed extract.

### Ki-67 expression in HepG2 cells after the treatment with the most powerful anticancer Annona extract

Figure 5C shows the immunohistochemistry for the proliferation marker (Ki-67) of HepG-2 cell line after treatment for 48 h with *Annona b.* and *Annona h.* seed extracts. These extracts were able to reduce Ki-67 level as indicated by increased purple color-stained cells (particularly in *Annona b*-treated HepG-2 cells) in comparison with brown color-stained untreated control cells (Fig. 5C-I). Figure 5C-II reveals that the Ki 67 level (2.51 ± 0.39%) was lower with *Annona b.* seed extract treatment than with *Annona h.* seed extract treatment HepG-2 cells (21.06 ± 1.93%). The potent inhibitory effect of seed extract of *Annona b*. on Ki-67 may attribute to their stronger apoptotic activity (Figs. 3B and 4) that was confirmed by higher percentage of the annexin-stained HepG2 population (50.14%) and reddish fluorescence-stained nuclei compared to 32.67% apoptosis and yellowish-orange stained nuclei in the case of *Annona* h. Additionally, the apoptosis-dependent anticancer effect of *Annona b*. seed was validated by up-regulation of p53 expression by sevenfold while it was twofold in the case of *Annona h.* seed-treated HepG2 cell line (Fig. 5B).

## Material and methods

### Collection of plant material

Fruits of *A. squamosa* (Balady) (*Annona b.*) and the cultivar Abdel Razik (a hybrid of two species: *Annona cherimola* × *A. squamosa*) (*Annona h*.) were purchased from Mansheya Market, Alexandria, Egypt. The fruits were identified and authenticated by Prof. Atef M. Ibrahim at the Pomology Department, Faculty of Agriculture, Alexandria University. After identification, the plant material was processed for extraction. The collection of Annona Fruits comply with relevant institutional, national, and international guidelines.

### Preparation of extracts

Peel, seeds, and pulp samples were oven-dried at 45 °C. The dried samples were ground separately into powder and stored at 4 °C until further use. The powdered samples were extracted with 1:20 w/v of distilled water and then heated at 70 °C for 30 min with stirring. The extracts were centrifuged at 10,000 × *g* for 25 min. then filtered, lyophilized, and stored in a freezer at − 20 °C until further use.

### Chemical analysis

Crude protein content determination, in dried powder form, was conducted using the Kjeldahl procedure according to Nelson and Sommers^52^. The crude fat and fiber contents were estimated in accordance with Folch et al.^53^, and Prosky et al.^54^, respectively. The mineral content, including Na, K, Ca, Mg, Cu, Mn, Zn, and Fe, was measured by microwave plasma atomic emission spectrometry (MP-AES) (Agilent 4100 MP-AES, USA), according to AOAC^55^. Phosphorus content was determined spectrophotometrically^56^. The mineral content was expressed as mg/kg of dry weight (dw). All measurements were carried out in triplicate.

### Phytochemical content

The total phenolic content was determined using Folin-Ciocalteu reagent, as described by Dewanto et al.^57^. The total flavonoid content of the extracts was quantified by a colorimetric method described by Sakanaka et al.^58^. Sulfated polysaccharides were measured by the toluidine blue assay as described by Albano and Mourao^59^. The triterpenoid content was assessed colorimetrically via reaction of the triterpenoids with vanillin using ursolic acid as a standard^60^. The tannin content was determined colorimetrically following the method described by Price et al.^61^. All measurements were carried out in triplicate.

### HPLC analysis for phenolics

Quantitative analyses of the phenolic content of the water extracts of the *Annona h*. and *Annona b.* seed were carried out by following the procedure described by Tomaino et al.^62^ using an RP-HPLC system (Agilent1260; Santa Clara, CA, USA) at 284 nm wavelengths with a C18 column (aKinetex 5lJm EVO C18 106 mm × 4.6 mm, Phenomenex, USA) maintained at 35 °C. The values for the main phenolic compounds were expressed in mg/100 g.

### Evaluation of biological activities of Annona h. and Annona b. extracts

#### Antioxidant activities

Determination of reducing power and the antiradical potentials (DPPH, ABTS) of different Annona parts of *Annona h.* and *Annona b.* were used to evaluate their antioxidant effects. Evaluation of the antiradical effect of each extract was done by estimating the IC_50_ values (50% inhibitory concentration). The reducing power of the extracts was determined according to the method described by Yen and Duh^63^, absorbance was measured at 700 nm. The reducing power was expressed as ascorbic acid equivalents (ASE)/mg. ASE is the reducing power of 1 mg of sample, which is equivalent to the reducing power of 1 nmol of ascorbic acid. The DPPH assay was carried out according to the method described by Shimada et al.^64^, and the absorbance of the solution at 517 nm was noted. For the ABTS radical scavenging assay, the method described by Re et al.^65^ was adopted, and the absorbance was measured at 734 nm after 7 min. All experiments were carried out in triplicate.

#### Antimicrobial activities

The agar well diffusion method was used to measure the antimicrobial activity of the peel, seeds, and pulp extracts of *Ann. s.* and *Ann. h.* against various pathogenic strains (*Escherichia coli* BA 12,296, *Bacillus subtilis* DB 100 host were kindly obtained from Dr. Sameh E. Mohamed, City of Scientific Research and Technology Applications (SRTA- CITY), Alexandria, Egypt); *Candida albicans* ATCC MYA-2876, *Klebsiella pneumoniae* ATCC12296, *Salmonella senftenberg* ATCC 8400, and *Staphylococcus aureus* NCTC 10,788 were obtained from Microbial Resource Center (MIRCEN), Ain Shams Univ. (ASU), Hadak Shobura, Cairo, Egypt. Antibacterial activity was evaluated by measuring the diameters (mm) of the inhibition zones using a caliper. The tests were performed in triplicate^66,67^.

### Determination of safe dose on the proliferation of normal human cells

Extract cytotoxicity was investigated using human lung fibroblast WI-38 cells (American Type Culture Collection (ATCC), USA) grown in DMEM (Lonza, USA) containing 5% fetal bovine serum (GIBCO, USA) by seeding as 3 × 10^3^ cells per well in a 96 well microtitre plate. The cells were treated with and without the serial extract dilutions after a 24 h incubation for cell attachment. The cytotoxicity assay was performed after 48 h, according to Mosmann^68^ using MTT reagent (Sigma, USA). The effective safe concentration (EC_100_) value (at 100% cell viability) of each tested extract was estimated by Graphpad Instat software (version 6.01).

### In vitro anticancer assay

Four human cancer cell lines (ATCC, USA) were used for this investigation. A colon cancer line (Caco-2) and a prostate cancer line (PC3) were maintained as adherent cell cultures in DMEM medium (Lonza, USA), while the HepG-2 (liver cancer line) and MCF-7 (breast cancer line) were cultivated in RPMI 1640 medium. These two media are supplemented with 10% fetal bovine serum. After cell seeding and attachment, cells were treated with and without the serial dilutions of extracts for 48 h in 5% CO_2_ incubator at 37 °C. The sensitivity of tumor cells to different Annona part samples was assessed using MTT assay. The anticancer activity of each tested sample was expressed as an IC_50_ value that causes 50% death for cancer cells and estimated by Graphpad Instat software. The morphological changes of all used cell lines were investigated by phase contrast inverted microscope (Olympus, Japan) after Annona seed extract treatment.

### In vitro detection of apoptosis-dependent anticancer effect using fluorescence microscope

Cancer cells were seeded in six well plates and incubated for cell attachment in 5% CO_2_ incubator for 24 h. Then the cancer cell lines (HepG-2, Caco-2, MCF-7, and PC-3) were treated with the most active part of *Annona h.* and *Annona b.* (seed extracts) for 48 h. After washing the cells with phosphate buffer saline (PBS), the cells were stained with dual fluorescent nuclear dye of EB/AO (Sigma, USA). The untreated and treated cancer cells were investigated using a fluorescence phase-contrast microscope (Olympus, Japan).

### Flow cytometry analysis

For flow cytometric detection, the untreated and treated human cancer cell lines (MCF-7, HepG-2, and Caco-2) were harvested by trypsinization then washed and resuspended in PBS. The mechanism of cancer cell death was analyzed by staining cells with fluorescein isothiocyanate (FITC)-annexin V and PI (Sigma, USA) for 15 min in the dark. Cells were then washed with PBS, fixed with 4% paraformaldehyde for 10 min and resuspended in PBS. Cell death rates were detected by flow cytometry (Partec, Germany) using a FITC signal detector (FL1) for annexin-stained apoptotic cells and the phycoerythrin emission signal detector (FL2) for PI-stained necrotic cells (Ex = 488 nm; Em = 530 nm).

### Real time PCR analysis for p53 and Bcl-2 expression

Quantitative gene expression of p53 and Bcl-2 were determined in cancer cells after treatment with *Annona b. and Annona h.* seed extracts separately. Total RNAs of all treated and untreated samples were extracted using a Gene JET RNA Purification Kit (Thermo Scientific, USA). The purity and the quantity of extracted RNA samples were determined using a UV-spectrophotometer, and cDNA was synthesized from mRNA by reverse transcriptase-polymerase chain reaction (RT-PCR), using a cDNA Synthesis Kit (Thermo Scientific, USA). Real time PCR was performed by amplification of cDNA for each sample using the following primers (Forward/Reverse) were 5′-TAACAGTTCCTGCATGGGCGGC-3′/5′-AGGACAGGCACAAACACGCACC-3′ and 5′- TCCGATCAGGAAGGCTAGAGTT-3′/5′-TCGGTCTCCTAAAAGCAGGC**-**3′ for p53 and Bcl-2, respectively. In addition, the following primers (5′-GTGGGCCGCTCTAGGCACCAA-3′/5′- CTCTTTGATGTCACGCACGATTTC-3′) were used for detecting the β- actin housing gene. The 2^−ΔΔCT^ equation was used to estimate changes in gene expression for p53 and Bcl-2 before and after treatment of cancer cells with Annona extracts.

### Immunohistochemical detection of proliferation marker (Ki-67)

After trypsinization, the untreated and treated HepG-2 cells were centrifuged and washed with PBS buffer and then 10% formalin in PBS was added to cell pellets. The fixed cell specimens were dehydrated in ascending grades of alcohol and immersed in xylene for one hour (three times), followed by impregnation in melted paraffin to form solid paraffin blocks. Next, a rotator microtome was used to cut each block into 3–5 μm thick sections transferred onto positively charged slides. Slides were dried at 60–70 °C for 1–2 h then dewaxed by immersion in xylene three times for 5 min and rehydrated in descending grades of ethanol. After that, slides were incubated in 3% H_2_O_2_ for 10 min, then washed in PBS buffer twice for 3 min and placed in 10 mM citrate buffer (pH 6), followed by heating at 60 °C for 10–20 min. After cooling and washing in PBS, the slides were separately soaked overnight in primary antibody (anti-Ki-67). Slides were washed in PBS, covered with biotinylated goat anti-polyvalent secondary antibody for 10 min, and then streptavidin peroxidase was added. After 10 min, the substrate of the secondary antibody (3,3′-diaminobenzidine) was added followed by washing in PBS and placed in a hematoxylin bath for 1–4 min. This was followed with a PBS wash (1 min) and then water (3 min). The percentage of immunostained cells was evaluated by imaging analysis software cellSens (CellSens Dimension 1.12) using a phase-contrast microscope (Olympus, Japan)^69^.

### Statistical analysis

Data were expressed as mean ± standard error of the mean by the multiple comparison one-way analysis of variance (ANOVA) and Tukey’s test using the SPSS16 software program with probability (*p*)- values < 0.05 considered statistically significant.

## Conclusion

Nutritional composition analysis of two Annona species peels, seeds, and pulp containing relatively high contents from protein, fat, fibers, carbohydrates, and good mineral quantity was done. The high potassium and calcium levels mean the species parts may be used for food supplementation, to increase the quality of food. Based on the current data the significantly high levels of phenolic compounds in *Annona* seeds, exhibited very high antioxidant potential compared to the peels and pulp. The polyphenol compounds in Annona seeds might induce effective bactericidal action against foodborne pathogens. The polyphenol rich extracts, probably o- Coumaric acid (49.02 mg/100 g), may be responsible for their apoptosis-dependent anticancer behavior against (Caco-2), prostate (PC3), liver (HepG-2), and breast (MCF-7) cancer cell lines. This is the first report on the possible molecular mechanism of action Annona *b.* seed extract has on cancer cell proliferation. Additional investigations will be required to characterize the bioactive compounds that may be responsible for the observed activities of the Annona seed to determine the potential mechanisms that make it an anticancer drug. Furthermore, Annona seed extract could be used in other food application and the manufacturing of nutraceuticals.

## Data Availability

All data generated or analyzed during this study are included in this published article.

## Abbreviations

GAE: Gallic acid
QE: Quercetin
RP-HPLC: Reversed-phase-High-performance liquid chromatography
DPPH: 2,2-Diphenyl-1-picrylhydrazyl
ABTS: 2,2′-Azino-bis(3-ethylbenzothiazoline-6-sulfonic acid
